# Chlorate of Potash in the Hæmorrhagic Diathesis

**Published:** 1880-05

**Authors:** A. Harkin

**Affiliations:** Belfast


### CHLORATE OF POTASH IN THE HÆMORRHAGIC DIATHESIS.

BY A. HARKIN, M. D., BELFAST.

Chlorate of potash, which is prescribed by the profession for a variety of diseases—such as scarlatina, throat-affections, low fevers, blood-poisoning, etc.—has qualities deserving a much wider application; and will yet, in the opinion of the writer, founded on extensive experience, be recognized as a potent remedy in the treatment of maladies depending on suboxidation, on defective nutrition, secretion, excretion, aeration, and molecular metamorphosis. Being mainly composed of oxygen and potassium, each of which is essential to the genesis of healthy blood, its chemical properties commend it to our consideration. In the hæmorrhagic diathesis, which is characterized by a diminished proportion of fibrin, a soft clot, an absence of the buffy coat, accompanied with a delicacy of structure in the capillaries and minute vessels, a remedy is required that shall increase the fibrin, add to the plasticity and chemico-vital elements of the blood and restore its coagulating power, as well as the contractile action of the capillaries; and thus destroy the dyscrasies, in which a slight wound may lead to excessive hæmorrhage, a trifling contusion to extensive extravasation. That this salt, whether given alone or in combination with iron, possesses the very desirable property of controlling the various developments of the hæmorrhagic diathesis, and that its persevering administration will neutralize the constitutional taint on which these ailments depend, Dr. Harkin hoped to establish by the relation of satisfactory cases, selected from an experience of its value extending over more than twenty years' observation. He generally ordered the medicine in the form one ounce of a saturated solution three times daily—one ounce of the salt to a pint of water; and, if iron be required, an addition of one drachm of the muriatic tincture to the solution completes the mixture.

Administered in this proportion, Dr. Harkin has had the greatest satisfaction in the treatment of epistaxis; in hæmophilia; in hæmorrhage from the bowels, from the kidneys, from the lungs, from the stomach; in menorrhagia; in scurvy; and in purpura hæmorrhagica.—*British Medical Journal.*

